# Sustaining Drug Discovery Amid the Limits of Alzheimer's Disease Immunotherapies

**DOI:** 10.1523/ENEURO.0112-26.2026

**Published:** 2026-07-23

**Authors:** Amina McDiarmid

**Affiliations:** Therapeutic Discovery, Institute of Genetics & Cancer, University of Edinburgh, Edinburgh EH4 2XU, United Kingdom

**Keywords:** Alzheimer’s disease, drug discovery, immunotherapy, neuroscience, novel treatments, translational science

## Abstract

Accumulation of insoluble extracellular amyloid-β (Aβ) plaques and intraneuronal fibrillary hyperphosphorylated tau fibrils in the brain are widely believed to contribute to the progressive neurodegeneration and neuronal loss characteristic of the Alzheimer’s disease and the associated dementia. Anti-amyloid immunotherapies that reduce cerebral Aβ burden in affected individuals have been approved based on biological outcomes from clinical trials. However, the extent to which Aβ clearance translates into improved meaningful clinical, cognitive, and functional benefit versus risk and cost is unclear based on available data. Here, the critical barriers that limit the impact of anti-amyloid immunotherapies, including aducanumab, lecanemab, and donanemab, are discussed. Treatment slows cognitive decline by ∼20–30% in certain patient subsets, but absolute improvements in cognitive and functional outcomes remain modest. Independent analyses of how treatment impacts health span suggests statistically significant trial results may not translate into significant real-world benefit. Response is most significant in cases of early-stage disease where levels of copathology tau are low. Edema and brain hemorrhage occur frequently, particularly in carriers of *APOE-e4* alleles, an established genetic risk factor for Alzheimer's disease, raising safety concerns which led to some regulators banning treatment in this patient group. Strict clinical trial eligibility criteria, high treatment costs relative to patient benefit, intensive during-treatment monitoring, and the absence of population-level screening programs further limit treatment accessibility and generalizability. Emerging evidence of accelerated brain atrophy and immunotherapy tolerance further complicates benefit–risk consultation. Sustaining drug- discovery pipelines by exploring combination therapies, tau-targeted approaches, drug repositioning, and novel small molecules through will be essential to provide comprehensive treatment options and personalized treatment plans for affected patients.

In Alzheimer's disease, amyloid-β (Aβ) plaques and Tau fibrils accumulate in the brain (Braak et al., 2006; Hampel et al., 2021). Affecting 55 million people globally, the associated dementia presents as a disabling decline in cognitive and executive function (Gauthier et al., 2022; GBD 2021 Nervous System Disorders Collaborators, 2024). Acetylcholinesterase and *N*-methyl-d-aspartate receptor inhibitors modify symptoms, but not progression or pathology. Anti-amyloid immunotherapies bind Aβ with high specificity, reducing cerebral Aβ burden via a cascade of immune-related events which represents a welcome shift to disease-modifying therapy. But treatment-related cognitive and functional outcomes are inconsistent, even for clinically approved anti-amyloid immunotherapies lecanemab and donanemab (Table 1). Treatment benefit may not outweigh risk in certain patient strata (Teipel et al., 2025). Initial results from clinical trials reported significant changes in cognitive and functional measures from baseline to trial endpoint, hinting that treatment may slow progression. Since then, independent analysis of currently available data suggests the significance of the result in trials does not translate to meaningful patient benefit in practice (Nonino et al., 2026). In their current state of development, anti-amyloid immunotherapies are limited by critical clinical, biological and economic barriers impacting treatment safety, tolerance, eligibility, generalizability and efficacy.

## Clinical Limits

Anti-amyloid immunotherapy trials quantified dementia outcomes using clinical assessments of cognitive and functional state, quantification of Aβ burden as the target pathology and biological response using blood-based and cerebrospinal fluid (CSF) biomarkers. Amyloid-β burden was measured using [^18^F]florbetapir positron emissions tomography (PET) with clearance indicated by values below standardized threshold of 24.1 centiloids (La Joie et al., 2025). The time to Aβ clearance (<24.1 centiloids) for aducanumab, lecanemab, and donanemab was 16–40, 12–24, and 6–20 months, respectively (Geerts et al., 2024; Table 1). In the head-to-head TRAILBLAZER-ALZ 4, donanemab outperformed aducanumab, the first-in-class amyloid-targeted antibody drug. Donanemab reduced median time to Aβ clearance by ∼6 months; however, cognitive endpoints were not measured (Table 2). Thus, aducanumab, lecanemab, and donanemab regulatory approval is supported by robust anti-amyloid efficacy. However, without between-trial standardization of other endpoint measures, interpreting treatment-related outcomes of broader neurobiological relevance is challenging.

**Table 1.** Summary of anti-amyloid immunotherapies for Alzheimer's disease in alphabetical order

Drug	Synonym	Target	Company	Status	Mechanism	Time to clearance	Delivery
Aducanumab	Aduhelm, BIIB037	Aβ aggregates/plaques	Biogen/Neurimmune	FDA approval via accelerated pathway in June 2021, discontinued in 2024 (futility), never approved by MHRA	Human monoclonal IgG1 antibody binding N-terminus of Aβ inhibiting aggregate formation	16–40 months (Geerts et al., 2024)	Intravenous infusion
Donanemab	Kisunla, N3pG-Aβ, LY3002813	Pyroglutamate Aβ in aggregates/plaques	Eli Lilly & Co	Full FDA approval in July 2024 and MHRA approval granted in October 2024	Humanized form of mE8-IgG2a targeted to multiple Aβ forms (Aβ3–42)	6–20 months (Geerts et al., 2024)	Intravenous infusion, subcutaneous injection
Gantenerumab	RO4909832, RG1450, R1450	Aβ fibrils	Hoffmann-La Roche/Chugai Pharmaceutical	Discontinued (futility)	Human IgG1 antibody binding both N-terminus and central epitopes in Aβ fibril conformation	Not published	Subcutaneous injection
Lecanemab	Leqembi, Lecanemab-irmb, BAN2401, mAb158	Soluble Aβ protofibrils	Eisai/Biogen, BioArctic	FDA approval by accelerated pathway in January 2023, upgraded to full approval in July 2023 and MHRA approval granted in August 2024	Humanized form of mAb158-IgG1 antibody selectively targeted to soluble Aβ fibrils	12–24 months (Geerts et al., 2024)	Intravenous infusion
Remternetug	LY3372993, N3pG-Aβ mAb	Pyroglutamate Aβ in plaques/aggregates	Eli Lilly & Co	In phase 3 clinical trials with no application for approval	Humanized monoclonal antibody targeting pyroglutamate Aβ	Not published	Subcutaneous injection
Solanezumab	LY2062430	Aβ peptides	Eli Lilly & Co	Discontinued	Humanized IgG1 monoclonal antibody binding monomeric soluble Aβ preventing fibril and subsequent plaque formation/development		Intravenous infusion, subcutaneous injection

Aβ, amyloid-β; MHRA, Medicines and Healthcare products Regulatory Agency; FDA, Food and Drug Administration; IgG, immunoglobulin type G.

**Table 2.** Endpoints measured in past/early phase studies and ongoing/active later phase clinical trials for approved anti-amyloid immunotherapies for Alzheimer's disease

Trial name (drug) phase	ClinicalTrials.gov identifier (status)	Primary endpoint	Other endpoint measures	Ref.
PRIME (aducanumab) Phase 1b	NCT01677572 (terminated after futility analysis for EMERGE and ENGAGE)	Number of participants experiencing adverse events	Change in cerebral Aβ from baseline (PET scan). Serum aducanumab concentration and incidence of anti-aducanumab antibodies from baseline	Chen et al. (2024) and Sevigny et al., (2016)
EMERGE (aducanumab) Phase 3	NCT02484547 (terminated after futility analysis)	Change in CDR-SB score from baseline in week 78	Change in ADAS-Cog13, MMSE, and ADCS-ADL-MCI score from baseline in week 78	Haeberlein et al. (2022)
ENGAGE (aducanumab) Phase 3	NCT02477800 (terminated after futility analysis)	Change in CDR-SB score from baseline in week 78	Change in ADAS-Cog13, MMSE, and ADCS-ADL-MCI score from baseline in week 78	Haeberlein et al. (2022)
EMBARK (aducanumab) Phase 3b	NCT04241068 (terminated by sponsor)	Incidence of events and serious adverse events, including those leading to study withdrawal and rate of ARIA-E and ARIA-H, serum anti-aducanumab antibodies	None	Castrillo-Viguera et al. (2021)
ENVISION (aducanumab) Phase 3b/4	NCT05310071 (terminated by sponsor)	Change in CDR-SB score from baseline in week 78	Change in ADAS-Cog13, MMSE, and ADCS-ADL-MCI score from baseline to week 78. Change in NPI-10 and GST from baseline to weeks 78 and 106. Change in CDR-SB in week 106 and change in Aβ and tau from baseline (PET scan) to weeks 78 and 104	Murphy et al. (2022)
TRAILBLAZER-ALZ (donanemab) Phase 2	NCT03367403 (completed)	Change in iADRS score from baseline in week 76	Change in ADAS-Cog13, CDR-SB, MMSE, and ACDS-iADL score from baseline in week 76. Change in Aβ and tau from baseline (PET scan) to week 76 and change in brain volume from baseline to week 76	Dickson et al. (2023), Mintun et al. (2021), Pontecorvo et al. (2022), Shcherbinin et al. (2022), and Zimmer et al. (2025)
TRAILBLAZER-ALZ 2 (donanemab) Phase 3	NCT04437511 (active not recruiting)	Change in iADRS score from baseline in week 76 in overall sample and in low-medium tau stratus	Change in ADAS-Cog13, CDR-SB, MMSE, and ACDS-iADL score from baseline to week 76 in overall sample and low-medium tau strata. Change in brain volume, Aβ, and tau from baseline (PET scan) to week 76 in overall sample. Serum donanemab concentration in week 16 to 20 and anti-donanemab antibodies from baseline to week 76	Lu et al. (2025); Sato et al. (2024), Sims et al. (2023), and Zimmer et al. (2025)
TRAILBLAZER-ALZ 3 (donanemab) Phase 3	NCT05026866 (active not recruiting)	Time to clinical progression as determined by cognitive composite score system CBR-GS from baseline to week 332	Change in pTau217, CBR-GS, ISLT, CPAL, iDSS, Category Fluency, CDR-SB, CFI, MoCA, MBI-C score up to week 332. Time to clinical progression as determined by CDR Memory Box/Judgement and Problem Solving Box. Serum concentration steady-state donanemab in week 76, and anti-donanemab antibodies from baseline to week 16	Yaari et al. (2025)
TRAILBLAZER-ALZ 4 (donanemab) Phase 3	NCT05108922 (completed)	Number of participants with AB-plaque negative PET scan (<24.1 centiloid) in overall sample and low-medium tau strata after 6 months	Mean absolute and percent change from baseline in cerebral Aβ signal from PET scan in donanemab versus aducanumab after 6, 12, and 18 months. Incidence of Aβ-plaque negative (<24.1 centiloid) in overall sample and low-medium tau strata after 12 and 18 months	Pain et al. (2023) and Salloway et al. (2023, 2025)
TRAILBLAZER-ALZ 5 (donanemab) Phase 3	NCT05508789 (recruiting)	Change in iADRS score from baseline in week 76 in low-medium or no-very low tau strata	Change in cerebral Aβ from baseline, iADRS and anti-donanemab antibodies from baseline to week 76 in overall population. Change from baseline ADAS-Cog13, CDR-SB, MMSE, and ACDS-iADL score in low-medium/no-very low tau strata or overall population to week 76. Serum concentration steady-state donanemab in week 76	–
TRAILBLAZER-ALZ 6 (donanemab) Phase 3b	NCT05738486 (active not recruiting)	Percentage of participants with ARIA-E in week 24	Incidence of ARIA-E/ARIA-H in week 52 and week 76 and ARIA-H in week 24. Change in cerebral Aβ from baseline to week 76. Serum donanemab concentration from baseline to week 76 and presence of anti-donanemab antibodies to week 91	Wang et al. (2025)
Study 201 (lecanemab) Phase 2	NCT01767311 (completed)	Change from baseline in ADCOMS score at 12 months; recording of adverse, and serious adverse, events	Change in CDR-SB, ADAS-Cog13 score and cerebral Aβ from baseline at 12 and 18 months, and brain volume, ADCOMS, CSF Aβ, t-Tau and p-Tau at 18 months. Change in hippocampal overall/bilateral volume at 6 and 12 months	Berry et al. (2023), Dhadda et al. (2022), and McDade et al. (2022)
Clarity AD (lecanemab) Phase 3	NCT03887455 (active not recruiting)	Change in CDR-SB at 18 months	Change in cerebral Aβ from baseline at 18 months. Change in ADAS-cog-13, ADCOMS, ACDS MCI-ADL at 18 months	van Dyck et al. (2023)
AHEAD 3–45 Study (lecanemab) Phase 2 and 3	NCT04468659 (active not recruiting)	Change in PACC5 score from baseline at 216 weeks (A45 trial). Change in cerebral Aβ from baseline (PET scan) at 216 weeks (A3 trial)	Change in cerebral Aβ and tau (PET scan) from baseline at weeks 96 and 216. Change in SFI score from baseline at 216 (A45 trial). Change in cerebral tau at week 216	Rafii et al. (2023)
DIAN-TU-001 (lecanemab and E2814) Phase 2 and 3	NCT05269394 (active not recruiting)	Change in cerebral tau (PET scan) from week 24 to 104 and 208 in the cohort with symptoms	Change in cerebral Aβ in weeks 0–24, CDR-SB in weeks 24–208, CSF neurofilament light from week 24 to week 104 and 208 in symptomatic group. Change in CSF pTau217/total tau in weeks 0–52 and 0–204 and 208. Change in CSF NFL light in week 82 to 104 and 208 in the asymptomatic group.	Wildsmith et al. (2025)

In TRAILBLAZER ALZ and TRAILBLAZER-ALZ 2, 4, and 5, [^18^F]flortaucipir PET imaging was used to determine cerebral tau burden and to stratify participants according to disease stage. Individuals were classified based on Alzheimer's disease-signature weighted standardized uptake value ratio (SUVR) thresholds, with low-medium tau defined as SUVR 1.10–1.46 and high tau by SUVR >1.46 (Shcherbinin et al., 2022; Sims et al., 2023). Tau, a clinical hallmark of Alzheimer's disease, spreads along clearly defined neuroanatomical pathways which correlate robustly with disease progression (Braak et al., 2006). Response to anti-amyloid drugs depends on an initially low tau burden TRAILBLAZER ALZ (Sims et al., 2023). Indeed, tau-related biomarkers such as plasma concentration of phosphorylated tau species pTau217 or pTau181 are increasingly used as proxy measures for central tau burden demonstrated by the fact later trials measured and reported changes in these biomarkers as secondary endpoints (Table 2). Result from these trials suggest anti-amyloid treatments may have indirect, downstream effects on tau pathology which could be integral to the drug's mechanism of action given that Braak stage may more accurately predict disease severity relative to cerebral Aβ burden. Biomarker-based stratification may also be an important step in patient selection, creating a barrier to treatment for a subset of Alzheimer's disease cases in which high tau is indicated but also increasing costs to determine patient eligibility.

Primary and secondary cognitive and functional outcomes were not consistent across trials. Most trials adopted a multiparametric approach, combining multiple cognitive and functional scales to monitor dementia progression (Table 2). Lecanemab (Study 201 and Clarity AD) and aducanumab (EMERGE and ENGAGE) trials primarily employed the Clinical Dementia Rating – Sum of Boxes Scale and, in parallel, measured ADAS-cog13 and ADCS-ADL-MCI scores (Table 2). Donanemab trials TRAILBLAZER-ALZ, ALZ 2, and ALZ 5 integrated the iADRS with Mini-Mental State Examination (MMSE) and a battery of cognitive scores including a modified assessment programed for prodromal cohort selected for TRAILBLAZER ALZ 3. There exists no universally accepted measure of, or threshold for, treatment-related cognitive benefit (Stoeckel et al., 2025). Thus, determining a robust association between anti-amyloid immunotherapy treatment and improved cognitive function is challenging due to inconsistencies in measurement and reported outcomes between and within trials to date.

Lecanemab treatment delayed cognitive decline by ∼30% across two trials and multiple cognitive/functional endpoints (Table 3). Donanemab slowed functional/cognitive decline by ∼22% in TRAILBLAZER-ALZ 2 (Sims et al., 2023; Sato et al., 2024; Lu et al., 2025; Zimmer et al., 2025) perhaps due to increased power relative to TRAILBLAZER ALZ (Table 3), in which minor changes to some minor functional endpoints were reported (Mintun et al., 2021; Shcherbinin et al., 2022). Aducanumab also slowed decline by ∼22% in a Phase Ib long-term extension (Chen et al., 2024), but conflicting evidence from concurrent trials EMERGE and ENGAGE halted further trials EMBARK and ENVISION (Haeberlein et al., 2022; Chen et al., 2024; Table 3). Reports from clinical trial endpoints suggests a slowed decline in episodic memory, orientation to time/date, judgment, and day-to-day tasks (such as self-care), but decline in complex multitasking, working memory, language skills (such as word finding and comprehension) and spatial praxis remained statistically unaffected (Sims et al., 2023). Furthermore, anti-amyloid immunotherapy clinical trials compared a percentage change from baseline to measure progression which may overestimate treatment-related cognitive benefit in the lived experience of a person affected by Alzheimer's disease.

**Table 3.** Summary of outcomes of key past/early phase studies and ongoing/active later phase clinical trials for approved anti-amyloid immunotherapies for Alzheimer's disease

Trial name (drug)	Design and dose	Participant number and status	Key outcomes	Ref.
PRIME (aducanumab)	Monthly intravenous infusions at high (10 mg/kg) or low dose (1 mg/kg, 3 mg/kg, or 6 mg/kg) for 52 weeks with LTE of further 112 doses	197 prodromal or mild AD cases (125 completed)	No ARIA in placebo groups. ARIA-E most frequent type of adverse reaction. Time- and dose-dependent reduction in amyloid PET with negative scan in high-dose group after 54 weeks of treatment. Trend for slowed decline as determined by CDR-SB in treatment groups (*p* = 0.07) after 1 year	Chen et al. (2024) and Sevigny et al. (2016)
EMERGE (aducanumab)	Monthly intravenous infusions at high (10 mg/kg) or low dose (3 mg/kg or 6 mg/kg) for 76 weeks	1,643 early/mild AD cases	Reduction in amyloid PET. Cognitive decline slowed by 22% in high-dose active treatment group after analysis of data from first 49% of participants	Haeberlein et al. (2022)
ENGAGE (aducanumab)	Monthly intravenous infusions at high (10 mg/kg) or low dose (3 mg/kg or 6 mg/kg) for 76 weeks	1,653 (early/mild AD)	Reduction in amyloid PET. Cognitive decline slowed by 15% favoring placebo after analysis of first 57% of participants	Haeberlein et al. (2022)
EMBARK (aducanumab)	Monthly 10 mg/kg for 100 weeks after initial titration-LTE additional 52 weeks	1,696 (early/mild AD)	None (trial terminated without reporting of results)	Castrillo-Viguera et al. (2021)
ENVISION (aducanumab)	Monthly 10 mg/kg for 78 or 106 weeks	1,027 (early/mild AD)	None (trial terminated without reporting of results)	Murphy et al. (2022)
TRAILBLAZER-ALZ (donanemab)	Titration from 700 mg intravenous infusion monthly after 12 weeks to 1,400 mg monthly for 60 weeks	272 (early/mild AD)	Reduction in amyloid PET, plasma pTau217, and GFAP. No change in plasma NFL or Aβ. Primary and most other endpoints not met. Higher sustained executive function (tasks associated with daily living) in active treatment group as determined by iADRS composite scoring. Time-component scores reported slowed progression of 5.2–5.3 months	Dickson et al. (2023), Mintun et al. (2021), Pontecorvo et al. (2022), Shcherbinin et al. (2022), and Zimmer et al. (2025)
TRAILBLAZER-ALZ 2 (donanemab)	Titration from 700 mg intravenous infusion monthly after 12 weeks to 1,400 mg monthly for 60 weeks	1,736 early/mild AD cases (1,320 complete)	Disease progression slowed based on iADRS score from baseline by 22%. Delayed progression by 4.46 months based on iADRS and 7.53 based on CDR-SB. Reduction in cerebral Aβ (PET scan). Whole brain and hippocampal volume decreased, ventricular volume increased. Aβ clearance in 1/3 of participants	Lu et al. (2025); Sato et al. (2024), Sims et al. (2023), and Zimmer et al. (2025)
TRAILBLAZER-ALZ 3 (donanemab)	Titration from 700 mg intravenous infusion monthly after 12 weeks to 1,400 mg monthly for 320 weeks	2,996 (prodromal AD)	Use of pTau217 to screen for preclinical AD supports identification of patients with early pathological hallmarks (amyloid levels 60–70 centiloids) but before symptom onset. Trial ongoing, no results reported yet	Yaari et al. (2025)
TRAILBLAZER-ALZ 4 (donanemab)	Titration from 700 mg intravenous infusion monthly after 12 weeks to 1,400 mg monthly for 6 months head-to-head with aducanumab	148 (early/mild AD)	Number of participants with amyloid negative PET scan was higher in donanemab treatment group (70% vs 43% in aducanumab group). Time to reach Aβ negative PET scan was reduced in donanemab treatment group (median time to <24.1 centiloids ∼1 year) compared with aducanumab treatment group (median time to <24.1 centiloids ∼1.5 years)	Pain et al. (2023) and Salloway et al. (2023, 2025)
TRAILBLAZER-ALZ 5 (donanemab)	Titration from 700 mg intravenous infusion monthly after 12 weeks to 1,400 mg monthly for 64 weeks	1,500 (early/mild AD)	Outcomes not yet reported	–
TRAILBLAZER-ALZ 6 (donanemab)	Titration from 350 mg intravenous infusion in week 1 to 700 mg in week 4, 1,050 mg in week 8 to 1,400 mg in week 12 (placebo weeks 2, 6, 10, and 14) then 1,400 mg dose monthly for 12 weeks. Compared dose-skipping, standard titration (see design for TRAILBLAZER-ALZ 2–5) and max concentration regimen	1,175 (early/mild AD)	Modified titration reduced rate of ARIA-E to half that of original regimen while retaining equivalent efficacy in amyloid clearance. Rate of side effects/adverse reactions unchanged by modified protocol	Wang et al. (2025)
Study 201 (lecanemab)	Fortnightly (2.5 mg/kg, 5 mg/kg, or 10 mg/kg) or monthly infusions or fortnightly infusions for 18 months (with LTE to 60 months)	856 (early AD)	Reduction in Aβ cerebral plaque burden and slowed cognitive decline by 30–55% depending on outcome (ADCOMS, CDR-DB, and ADAS-cog13). Inverse correlation between cognition and plaque clearance	Berry et al. (2023), Dhadda et al. (2022), and McDade et al. (2022)
Clarity AD (lecanemab)	Fortnightly infusion with 10 mg/kg lecanemab for 18 months, some participants assigned to weekly 260 mg subcutaneous injection group	1,906 (early AD)	Fortnightly 10 mg/kg lecanemab dose reduced Aβ cerebral plaque burden and slowed cognitive decline by 27% based on CDR-SB (20–30% for ADCOMS, ADAS-cog13, and ACDS MCI-ADL)	van Dyck et al. (2023)
AHEAD 3-45 Study (lecanemab)	Fortnightly infusion with 10 mg/kg lecanemab for 216 weeks	1,400 (preclinical AD)	Results not yet reported	Rafii et al. (2023)
DIAN-TU-001 (lecanemab and E2814)		197 (individuals with ADAD mutations with and without symptoms)	Interim publication reports reduction in cerebral Aβ, similar to sporadic early stage cases in other clinical trials	Wildsmith et al. (2025)

Time-component analysis shows lecanemab and donanemab slow progression by 5–7 months (Dickson et al., 2023;  van Dyck et al., 2023). Treatment may prevent progression from mild to moderate and moderate to severe Alzheimer's disease (Atri et al., 2026; Burn et al., 2026). But an independent Cochrane review (Nonino et al., 2026) and Bayesian analysis (Teipel et al., 2025) suggests that when the data is taken together and exploratory statistical methods applied, treatment does not drive substantial clinical benefit despite statistically significant outcomes from individual clinical trial reports. In clinical practice, the MMSE is part of the Alzheimer's disease diagnostic pathway so is an important measure of clinical benefit. MMSE was commonly excluded from many trials as a prespecified endpoint, with many including it only as an exploratory analysis. If posttreatment absolute measures of cognitive function are compared as opposed to than relative values that express outcomes as a change from baseline, the effects are minimal.

In TRAILBLAZER ALZ 2, those who received donanemab had an absolute mean difference of +3.25 points on the 144-point iADRS (Sims et al., 2023). An absolute difference of 0.45 points in the 18-point CDR-SB scale was observed between active treatment and placebo control groups in Clarity AD trial in favor of lecanemab (van Dyck et al., 2023). Symptomatic treatment with cognitive enhancers donepezil, rivastigmine, memantine, or galantamine improves cognitive scores to a similar, if not greater, extent (Burke et al., 2023; Espay et al., 2024). After treatment with lecanemab, donanemab, or aducanumab, MMSE scores are 0.3–0.5 points higher than those who received placebo (Ebell et al., 2024). This incremental difference in absolute score sits within standard error margins for day-to-day testing (Feeney et al., 2016). There is no study that directly compares anti-amyloid immunotherapy outcomes with treatment as usual controls, so whether individuals who have received these treatments are improved relative to those on standard care plans is unclear.

Treatment response also varied between participant groups. Donanemab treatment delayed cognitive decline by 22.3% in high tau participants (a more advanced disease state), significantly less than a 35.1% delay in low-medium tau participants (Sims et al., 2023; Zimmer et al., 2025). Secondary analysis suggests reduced efficacy of donanemab and lecanemab in *APOE-e4* homozygotes (Qiao et al., 2023; Jeremic et al., 2025; Pereira da Silva et al., 2025; Teipel et al., 2025; Zimmer et al., 2025). Absence of clinical benefit was four times more likely in *APOE-e4* homozygotes (Teipel et al., 2025) who represent 15% of population-level cases and experience a >50% lifetime risk of Alzheimer's disease (Reiman et al., 2025; Williams et al., 2026).

Adverse reaction frequency increased in *APOE-e4* carriers (Table 4), with significant genome-wide association between *APOE-e4* genotype and amyloid-related imaging anomalies (ARIA), edema (ARIA-E) and hemorrhage (ARIA-H; Honig et al., 2024; Loomis et al., 2024; Shields et al., 2024). *APOE-e4* homozygotes are, therefore, not eligible for treatment in some areas (Commission authorises medicine for treatment of early Alzheimer’s disease, 2025). Overall, treatment-related adverse reactions, most commonly edema and hemorrhage, occurred in 30–80% of active treatment participants (Table 4; Lalli et al., 2021; Lowe et al., 2021; Swanson et al., 2021; McDade et al., 2022; Shcherbinin et al., 2022; Sims et al., 2023). Administering a treatment that carries risk of cerebral hemorrhage, which itself lacks effective treatment, is concerning. While we have some understanding of the acute adverse reactions to treatment based on clinical trial data, longer term adverse effects are becoming clearer. Significant anti-amyloid immunotherapy-induced accelerated brain atrophy has been reported and is associated with ARIA frequency during treatment (Alves et al., 2023). Thus, modest effects on cognitive decline from Phase 1 to 3 trials did not provide conclusive evidence that treatment benefit versus acute and chronic risk to brain health is related to meaningful, real-life impact on dementia progression.

**Table 4.** Efficacy, adverse reactions, and stratified effects for anti-amyloid immunotherapies

Trial name(s) (drug)	Stratified outcomes	Overall rate of side effects/adverse reaction	Stratified side effects/adverse reactions	References
PRIME (aducanumab)	Amyloid PET reduction and cognitive decline similar in mild and prodromal cases irrespective of ApoE genotype	∼50% experienced adverse reaction with 20% for ARIA-H and 40% for ARIA-E with some experiencing both. Infusion reactions most common side effect	ARIA-E more common in *APOE-e4* carriers (50%) versus noncarriers (8%), dose titration reduced increase, ARIA-E more common in high-dose group (41%)	Sevigny et al. (2016) and Chen et al. (2024)
EMERGE and ENGAGE (aducanumab)	Amyloid PET reduction and cognitive decline similar in ApoE-e4 carriers versus noncarriers	∼35% experienced ARIA-E and ∼20% reported ARIA-H	ARIA-E more common in *APOE-e4* carriers (40%) versus noncarriers (18–23%), ARIA-H more common in ARIA-E (20% of ARIA-H in those reporting ARIA-E compared with 6–9% without ARIA-E). GWAS analysis reports association of *APOE-e4* variant with serious side effects	Haeberlein et al. (2022) and Loomis et al. (2024)
TRAILBLAZER ALZ (donanemab)	Correlation between improved iADRS and reduction in cerebral Aβ burden in APOE-e4 homozygotes only. Younger participants with less severe disease showed best iADRS-related outcomes	ARIA-E and ARIA-H 27–31% of participants receiving donanemab. Serious and symptomatic cases of ARIA-E affected 1.5 and 5.8% of those in active treatment groups, respectively	ARIA-E increased in with APOE-e4 allele count, rate of ARIA-H, high blood pressure, and high cerebral Aβ burden at baseline. Risk of ARIA decreased with use of antihypertensive drugs	Greenberg et al. (2024)
TRAILBLAZER ALZ 2 (donanemab)	Amyloid PET reduction and cognitive decline similar in ApoE-e4 carriers versus noncarriers. Slowed cognitive decline confirmed in LTE of trial, with best outcomes reported for those treated at earlier stages	Infusion reaction and ARIA-E/ARIA-H in 8.7 and 24% of participants receiving active treatment, respectively, treatment-related deaths occurred in 3 participants receiving donanemab compared with 1 death in placebo	ARIA-E occurred more frequently in APOE-e4 homozygotes (∼40%) relative to heterozygotes (∼25%) and noncarriers (∼15%), and ARIA-H also more frequent in homozygotes (53%) compared with heterozygotes (18%) confirmed in open-label addendum	Sims et al. (2023) and Zimmer et al. (2025)
TRAILBLAZER ALZ 3 (donanemab)	Group with CDR-GS scores of 0 mean Aβ burden 63.2 centiloids, whereas group CDR-GS of 0.5 mean Aβ burden was 70.7 centiloids. Elevated plasma p-Tau217 in 15.1% of CDR-GS = 0 group, compared with 26.3% in CDR-GS = 0.5	No results reported yet	No results reported yet	Yaari et al. (2025)
TRAILBLAZER ALZ 4 (donanemab)	In the group with low-medium tau (considered early stage), 80.0% of participants Aβ burden was <24 centiloids by 12 months after treatment with donanemab compared with 9.6% of low-medium tau participants treated with aducanumab	ARIA-E in aducanumab-treated participants (35%) compared with donanemab-treated participants (24%)	ARIA-E or ARIA-H highest in aducanumab-treated APOE-e4 homozygotes (70%) compared with those who were donanemab-treated (44.4%). ∼35% of noncarriers treated with aducanumab were affected with ARIA-E or ARIA-H, as did 35% of APOE-e4 heterozygotes in aducanumab or donanemab treatment groups irrespectively	Pain et al. (2023)
TRAILBLAZER ALZ 6 (donanemab)	Modified titration associated with decrease in ARIA risk	ARIA-E and ARIA-H occurred in ∼24% on standard regimen, reduced to ∼15% in adjust titration regimen	Risk decreased from almost 60 to 19% on standard regimen versus modified titration, respectively, representing a ∼67% decrease. ARIA in APOE-e4 heterozygotes reduced by ∼38 and ∼12%, respectively	Wang et al. (2025)
Study 201 (lecanemab)	Initially reported a greater reduction in cognitive decline in APOE-e4 carriers	Infusion reactions reported in 19.9% of participants. ARIA-E and ARIA-H reported in 9.9 and 6.2–10.6% of treated participants, respectively	APOE-e4 homozygotes had 10 times greater risk of ARIA, with 50% of APOE-e4 homozygous participants reporting ARIA-E or ARIA-H compared with 5.1% of APOE-e4 heterozygotes and 8.0% in noncarriers	Honig et al. (2024)
Clarity AD (lecanemab)	APOE-e4 homozygotes had detectable slowing of cognitive decline in 2 out of 4 outcome measures (ADCOMS and ADAS-cog13) whereas noncarriers showed significant change in ADCOMS, CDR-SB, ADAD-cog13, and ACDS MCI-ADL	Infusion-related reactions, ARIA-E and ARIA-H occurred in 26.4% versus 7.4%, 12.6% versus 1.7%, and 17.3% versus 9.0%, respectively. Adverse events in lecanemab-treated participants 44.4%, but 11.3% of placebo	Higher rate of withdrawal in active treatment groups (6.9% vs 2.9%). Clinical benefit for APOE-e4 homozygotes and carriers impacted by higher rates of adverse reaction (ARIA-E in 5.1% of noncarriers, but 10.9 and 32.6% APOE-e4 heterozygotes and homozygotes, respectively)	van Dyck et al. (2023)
DIAN-TU-001 (lecanemab and E2814)	Faster time to amyloid clearance (<24 centiloids) in aged patient groups	80.4% of experienced adverse events related to treatment	Infusion reactions 11.3%, with ARIA-E and ARIA-H experience in ∼20% of ADAD carriers. 6.2% of experienced serious adverse events	Wildsmith et al. (2025) and Zhou et al. (2025)

Aβ, amyloid-β; AD, Alzheimer's disease; ADCOMS, Alzheimer's Disease Composite Score; ADAS-cog, Alzheimer's disease Assessment Scale – Cognitive Subscale; ADAD, Autosomal dominant Alzheimer's disease; ADCS-ADL, Alzheimer's Disease Cooperative Study Activities of Daily Living; ARIA, amyloid-related imaging anomaly; ARIA-E, amyloid-related imaging anomaly—edema; ARIA-H, amyloid-related imaging anomaly—hemorrhage; CDR-SB, Clinical Dementia Rating – Sum of Boxes; CDR-GS, Clinical Dementia Rating – Global Score; CFI, Cognitive Function Instrument; CPAL, Continuous Paired Associate Learning; CSF, cerebrospinal fluid; iADRS, Integrated Alzheimer's Disease Rating Scale; IDSS, Cogstate Intelligent Decision Support System; GFAP, glial fibrillary acid protein; GST, Global Statistical Test; ISLT, International Shopping List Test; LTE, long-term extension; MCI, mild cognitive impairment; MMSE, Mini-Mental State Examination; MoCA, Montreal Cognitive Assessment; NPI-10, Neuropsychiatric Inventory – 10; PACC5, Preclinical Alzheimer's Cognitive Composite 5; MRI, magnetic resonance imaging; PET, positron emission tomography.

Due to strict inclusion/exclusion criteria (Table 5), efficacy/safety in heterogeneous clinical populations is not clear from trial data (Rajan et al., 2025). Comorbidities, such as autoimmune disease, affect eligibility. Autoimmune disease, affecting 10% of the UK population (Conrad et al., 2023), is enriched in Alzheimer's disease cohorts (Wotton and Goldacre, 2017). Autoimmune biomarkers increase with age (Miller, 2023). Risk of immune-related adverse events in autoimmune condition cases is higher (Boland et al., 2020; Tison et al., 2022). Such individuals are excluded from clinical trials thus far. So too have individuals with past/present malignancy, cardiovascular disease, psychiatric diagnosis, medication use (psychotropic and/or anticoagulant and/or immunological), and neuroimaging anomalies (Claus et al., 2025; Jones et al., 2025; Table 5). Only 9–31% of Alzheimer's disease cases would be eligible for treatment under current appropriate use recommendations (Rosen and Jessen, 2025). A dearth of safety/efficacy data from broader patient groups limits generalizability of clinical trial outcomes, narrowing the window of indication.

**Table 5.** Inclusion/exclusion criteria for Phase 2/3 clinical trials of anti-amyloid immunotherapies for Alzheimer's disease

Trial name(s) (drug)	Key inclusion criteria	Key exclusion criteria
PRIME (aducanumab)	For prodromal cases: 24 ≤ MMSE≤30, 0.5 = CDR-SB, FCSRT≤27, spontaneous memory complaint. For mild AD cases: 19 < MMSE < 27, 0.5 < CDR-SB < 1, presentation of NIAAA core clinical criteria for AD, amyloid-positive PET scan, reliable informant/carer, consent to ApoE genotyping	MMSE > 19, no other health conditions (neurological or psychiatric)/TIA/seizure, cardiac conduction abnormalities or heart condition, brain MRI abnormalities, anticoagulant use or change to prescribed medicines within 4 weeks, brain micro- or macrohemorrhage <4, lunar/cortical infarct ≤1. For prodromal cases: no other cognitive impairment or dementia present
EMERGE and ENGAGE (aducanumab)	Mild/early AD cases: Amyloid-positive PET scan, 24 ≤ MMSE≤30, 0.5 = CDR-SB, objectively assessed cognitive impairment, reliable informant/carer, consent to ApoE genotyping	Neurological or psychiatric/TIA/seizure conditions, cardiac conduction abnormalities or heart condition, brain MRI abnormalities, brain hemorrhage or cerebrovascular abnormalities, impaired renal or liver function
TRAILBLAZER ALZ (donanemab)	Subjective or informant experience of progressive decline in memory function over >6 months and 20 ≤ MMSE score ≤28 at baseline or Aβ-positive PET scan within 6 months	Cardiac anomalies (e.g., long WT syndrome), treatment with acetylcholinesterase inhibitor and/or memantine (treatment as usual) for <2 months before recruitment and randomization. Neuroimaging anomalies detected on MRI
TRAILBLAZER ALZ 2 (donanemab)	Subjective or informant experience of decline in memory over >6 months and 20 ≤ MMSE score ≤28 at baseline or Aβ-positive PET scan within 6 months. Reliable study partner	Neuroimaging anomalies detected on MRI or by PET. Current treatment with immunotherapy (immunoglobulin G, IgG)
TRAILBLAZER ALZ 3 (donanemab)	Intact cognition as determined by telephone interview using TICS-M score. Presence of p-Tau consistent with that expected for individual with amyloid pathology. Reliable study partner to provide informed consent and inform on function/behavior. Adequate vision, hearing, and literacy skills base on neuropsychological assessment. Mandatory contraceptive use	No condition affecting cognition including mild cognitive impairment, dementia, or neurodegenerative disease. Cardiovascular, blood, liver, kidney, digestive, respiratory, hormonal, neurological, psychiatric, malignant, or immune-related disease. Life expectancy ≤5 years. Any drug allergy. MRI anomalies or MRI contraindications (i.e., claustrophobia). Recent treatment with passive, or any prior treatment with active, anti-amyloid immunotherapy. Prior or current use of medications used for cognitive impairment
TRAILBLAZER ALZ 4 (donanemab)	Subjective or informant experience of progressive decline in memory function over >6 months, Aβ-positive PET scan, score of 0.5 or 1 on CDR-GS and 20–30 on MMSE, consent to ApoE genotyping and reliable study partner to provide informed consent and inform on function/behavior based on frequent contact (>10 h per week) with participant. Adequate vision, hearing, and literacy skills base on neuropsychological assessment	Neurological illness affecting CNS (e.g., other form of dementia, neuroinflammation or infection, Parkinson's disease, concussion, transient ischemic attack, epilepsy, or seizures). Medical illness (e.g., cardiovascular, blood, liver, kidney, digestive, respiratory, hormonal, psychiatric, or immune-related disease or life expectancy <2 years). Exclusion determined case-by-case based in investigator assessment of potential of comorbidity to confound analysis. Drug allergy or sensitivity. Anticoagulant medication use. Prior anti-amyloid immunotherapy treatment
TRAILBLAZER ALZ 5 (donanemab)	Subjective or informant experience of progressive decline in memory function over >6 months, Aβ-positive PET scan, score of 0.5 or 1 on CDR-GS and 20–28 on MMSE, consent to ApoE genotyping and at least one reliable study partner to provide informed consent and inform on and be present for assessment of function/behavior. Must be in frequent contact (>10 h per week). Stable tau for >30 d prior	Presence of neurological illness affecting CNS (e.g., other form of dementia, neuroinflammation or infection, Parkinson's disease, concussion, transient ischemic attack, epilepsy, or seizures). Medical illness (e.g., cardiovascular, blood, liver, kidney, digestive, respiratory, hormonal, neurological, psychiatric, or immune-related disease or life expectancy <2 years). Malignancy (apart from certain skin and non-metastatic cancers) in the last 5 years. MRI or PET anomalies
TRAILBLAZER ALZ 6 (donanemab)	Subjective or informant experience of progressive decline in memory function over >6 months, Aβ-positive PET scan and 20–28 on MMSE	Medical or neurological illness (e.g., other dementia, neuroinflammation or infection, Parkinson's disease, concussion, TIA, epilepsy or seizures, cardiovascular, blood, liver, kidney, digestive, respiratory, hormonal, neurological, psychiatric, or immune-related disease or life expectancy <2 years). Malignant neoplasms in the last 5 years. Prior treatment with anti-amyloid immunotherapy
Study 201 and Clarity AD (lecanemab)	Subjects with probable AD after objective assessment with MMSE between 23 and 30, 0.5 or 1 in CDR score, Memory Box score ≥0.5, and age-adjusted WMS- IV LMII at baseline meeting NIA-AA clinical criteria for MCI due to AD. Subjective experience of decline in memory over 12 months. Aβ-positive PET scan or CSF sample. Informant/caregiver identified	BMI not between 17 and 35 inclusive at baseline. Women screened for possible pregnancy. Any neurological illness, transient ischemic attack or seizures. Geriatric Depression Scale ≥8 at baseline. MRI contraindications (i.e., pacemaker fitted, metal implants, claustrophobia). MRI anomalies. Cardiac conduction anomalies such as long QT syndrome. Use of certain medications, or unstable AD medication use in 12 weeks prior to study
AHEAD 3-45 Study (lecanemab)	Aβ-positive PET scan or CSF sample or risk factor (1st relative with dementia onset before 75 years/APOE-e4 carrier/homozygote). CDR score = 0 at baseline, ≤MMSE score ≥27 and WMS-R LM II ≥ 6. Reliable study partner. Compliant with experimental protocol. No blood test anomalies, i.e., platelet count <50,000, TSH higher than normal range, B12 below normal range, HIV-positive	Women screened for pregnancy not on effective contraception. Transient ischemic attack or seizures in 12 months up to study commencement. Psychiatric illness in 2 years up to study, including suicidal ideation (C-SSRS) in 6 months up to study start or in-patient treatment for psychiatric illness in 5 years. Malignancy (within 3 years) or immune-related disorder. Controlled substance use. Monoclonal antibody sensitivity, IgG or anti-amyloid immunotherapy, or experimental medicine treatment within 6 months
DIAN-TU-001 (lecanemab and E2814)	Confirmed of ADAD mutation. Within 10 years of predicted or actual age of onset. CDR score 0–1 inclusive. Reliable study informant/partner. Compliant with MRI, lumbar puncture and PET scan	Mandatory contraception for women of childbearing age and screened for pregnancy. Anticoagulant use. Psychiatric illness (suicide attempts ideation) in last 12 months. Anti-amyloid immunotherapy exposure medicine within 6 months of start. Malignancy within 3 years of study start

Aβ, amyloid-β; AD, Alzheimer's disease; ADCOMS, Alzheimer's Disease Composite Score; ADAS-cog, Alzheimer's Disease Assessment Scale – Cognitive Subscale; ADAD, Autosomal dominant Alzheimer's disease; ADCS-ADL, Alzheimer's Disease Cooperative Study Activities of Daily Living; ARIA, amyloid-related imaging anomaly; ARIA-E, amyloid-related imaging anomaly—edema; ARIA-H, amyloid-related imaging anomaly—hemorrhage; CDR-SB, Clinical Dementia Rating – Sum of Boxes; CDR-GS, Clinical Dementia Rating – Global Score; CFI, Cognitive Function Instrument; CPAL, Continuous Paired Associate Learning; CSF, cerebrospinal fluid; iADRS, Integrated Alzheimer's Disease Rating Scale; IDSS, Cogstate Intelligent Decision Support System; GFAP, glial fibrillary acid protein; GST, Global Statistical Test; ISLT, International Shopping List Test; LTE, long-term extension; MCI, mild cognitive impairment; MMSE, Mini-Mental State Examination; MoCA, Montreal Cognitive Assessment; NPI-10, Neuropsychiatric Inventory – 10; PACC5, Preclinical Alzheimer's Cognitive Composite 5; MRI, magnetic resonance imaging; PET, positron emission tomography.

## Economic Issues

High treatment cost is attributed to clinically intensive drug infusion regimens with neuroimaging, biomarker, and cognitive/functional monitoring. Lecanemab and donanemab do not sufficiently improvement quality and quantity of life relative to cost (Kmietowicz and Mahase, 2024; Mahase, 2025, 2024), with incremental gains of ∼0.1 quantity adjusted life years (QALYs; Haile and Lee, 2024) equating to one additional month lived in perfect health. Perhaps transitioning from administration by intravenous infusion to subcutaneous injection, as is currently being trialed for donanemab, gantenerumab, and remternetug (Table 1), will drop the economic and clinical burden associated with delivery tipping cost versus benefit analysis in favor of treatment (Tahami Monfared et al., 2025).

Clinical trials were limited to individuals with mild- or early stage disease (Claus et al., 2025; Jones et al., 2025), since they act to stabilize progression. Lecanemab and donanemab efficacy depends on early intervention (Lowe et al., 2021; Swanson et al., 2021; McDade et al., 2022; Sims et al., 2023; van Dyck et al., 2023). Yet, no national screening program exists to identify preclinical/prodromal cases.

## Biological Barriers

Immunotherapy tolerance is an emerging issue after high incidence of anti-drug antibodies was reported in trial participants (Mintun et al., 2021; Mullins et al., 2025). Alternative delivery, antibodies, and immunotherapies targeted to other pathologies are in development (e.g., tau-targeted immunotherapies; Table 6), paving the way for combined therapy (e.g., DIAN-TU-001). Developing both disease-modifying and symptomatic-targeted therapeutics will support future expansion of treatment options (Cummings et al., 2025), perhaps including MAPT- and APP-targeted anti-sense oligonucleotides (Table 7), opening possibilities for personalized/patient-centered treatment plans.

**Table 6.** Other anti-amyloid immunotherapies and tau-targeted therapeutics in development

Trial name (drug)	Treatment type	Trial name	Phase	ClinicalTrials.gov identifier	Target	Status	Company	Ref.
Remternetug	Subcutaneous immunotherapy	DIAN-TU-002	2 and 3	NCT06647498	Aβ	Recruiting	Washington University School of Medicine	-
		TRAILRUNNER ALZ 1	3	NCT05463731	Aβ	Active, not recruiting	Eli Lilly & Co	
		TRAILRUNNER ALZ 3	3	NCT06653153	Aβ	Active, not recruiting	Eli Lilly & Co	Kevin et al. (2025)
Gantenerumab	Intravenous immunotherapy	SKYLINE	3	NCT05256134	Aβ	Terminated	Hoffmann-La Roche	Bateman et al. (2022)
		GRADUATE I	3	NCT03444870	Aβ	Terminated	Hoffmann-La Roche	Bateman et al. (2023)
		GRADUATE II	3	NCT03443973	Aβ	Terminated	Hoffmann-La Roche	Bateman et al. (2023)
Solanezumab	Intravenous immunotherapy	A4	3	NCT02008357	Aβ	Completed	Eli Lilly & Co	Sperling et al. (2023)
		EXPEDITION-1 and 2	3	NCT05967351	Aβ	Enrolling by invitation	Sarepta Therapeutics	Doody et al. (2014)
		EXPEDITION-3	3	NCT01900665	Aβ	Terminated	Eli Lilly & Co	Honig et al. (2018)
Solanezumab and gantenerumab	Combined anti-amyloid immunotherapies	DIAN-TU-001	2 and 3	NCT01760005	Aβ	Active, not recruiting	Washington University School of Medicine	Bateman et al. (2025) and Wagemann et al. (2024)
ACI-35	Intramuscular immunotherapy	-	1b and 2a	NCT04445831	Tau	Completed	AC Immune SA	Sol et al. (2025)
Bepranemab	Immunotherapy	TOGETHER	2	NCT04867616	Tau	Completed	UCB Biopharma	Maguire et al. (2025)
JNJ-63733657 (posdinemab)	Immunotherapy	AUTONOMY	2	NCT04619420	Tau	Active, not recruiting	Janssen Research & Development	Wang et al. (2026)
E2814 (etalanetug)	Immunotherapy	DIAN-TU-001	2 and 3	NCT05269394	Tau	Active, not recruiting	Washington University School of Medicine/Eisai	Horie et al. (2025)
BIIB080	Injected DNA and RNA-based therapy	CELIA	2	NCT05399888	Tau	Active, not recruiting	Biogen	Ziogas et al. (2023)
LY3372689	Small molecule	PROSPECT-ALZ	2	NCT05063539	Tau	Completed	Eli Lilly & Co	Fleisher et al. (2025)

Aβ, amyloid-β.

**Table 7.** Small molecule and anti-sense oligonucleotide therapies in development for AD

Drug	Clinical strategy	Type	Phase	ClinicalTrials.gov identifier	Target	Mechanism of action	Status
ATH-1017	Neuroprotective, disease-modifying	Small molecule	2/3	NCT04488419	HGF/MET receptor	Enhance neurotrophic signaling	Terminated
Buntanetap	Neuroprotective with disease-modifying potential	Small molecule	2/3	NCT05686044	Protein synthesis	Inhibit translation of APP, tau, and a-synuclein	Completed
Hydralazine	Neuroprotective	Small molecule	3	NCT04842552	NRF2 pathway	Vasodilator, enhances autophagy and mitochondrial function	Unknown
Masitinib	Anti-inflammatory	Small molecule	3	NCT05564169	Tyrosine kinase inhibitor	Inhibits neuroimmune cell (mast cell and microglia) function	Not yet recruiting
Metformin	Metabolic regulation	Small molecule	2/3	NCT04098666	AMPK agonist	Insulin-sensitizing agent	Active, not recruiting
NE3107	Anti-inflammatory and metabolic regulation	Small molecule	3	NCT04669028	ERK/NFkB inhibitor	Brain-penetrant suppressor of inflammatory factors	Completed
Nilotinib	Neuroprotective	Small molecule	3	NCT05143528	Tyrosine kinase inhibitor	Brain-penetrant inhibitor of DDR1 and c-Abl, cell homeostasis	Unknown
Semaglutide	Neuroprotective with disease-modifying potential	Small molecule	3	NCT04777396 and NCT 04777409	GLP-1 agonist	Brain-penetrant regulator of glucose metabolism	Completed
Diranersen (formerly BIIB080/MAPTRx)	Disease-modifying	Anti-sense oligonucleotide	2	NCT05399888	MAPT mRNA	Reduce expression of tau by inhibiting protein synthesis	Active, not recruiting
ALN-APP	Disease-modifying	Anti-sense oligonucleotide	1	NCT05231785	Amyloid precursor protein mRNA	Reduce expression of APP, the upstream precursor of Aβ	Recruiting

Aβ, amyloid-β; AMPK, adenosine monophosphate-activate protein kinase; APP, amyloid precursor protein; ERK, extracellular signaling related kinase; GLP-1, glucagon-like peptide-1; HGF, hepatocyte growth factor; MET, c-MET gene; MAPT, microtubule-associated protein tau; mRNA, messenger ribonucleic acid; NFkB, nuclear factor kappa-light-chain-enhancer of activated B cells; NRF2, nuclear factor erythroid-factor 2-related factor 2.

Small molecules, with their diverse chemistry, offer opportunities to modify the alternative molecular/cellular mechanisms now known to contribute to Alzheimer's disease/dementia symptoms including, but not limited to, Aβ and tau pathology (Rawal et al., 2025; Table 7). Performing screens in human in vitro cell models to has been successful for other human neurodegenerative diseases, as shown by ezogabine's repurposing for amytrophic lateral sclerosis after discovery in patient stem cell lines (Kiskinis et al., 2014; Moakley et al., 2019; Wainger et al., 2021). Small molecules may also enhance immunotherapy efficacy/tolerance (Wang et al., 2024; Wu et al., 2024). Thus, similar approaches may yield successes for Alzheimer's disease.

## Sustaining Drug Discovery Pipelines for Alzheimer's Disease

Anti-amyloid immunotherapy may not translate to substantial improvements to quality or quantity of life. Drug resistance, cost versus benefit, and restricted therapeutic indication prevent widespread adoption of currently available anti-amyloid immunotherapies. Sustaining modern drug discovery pipelines is, therefore, essential to tackle attrition during preclinical/early phase clinical testing. Delivery of disease-modifying immunotherapies provides hope but does not, completely, meet the demand for safe/effective treatment plans in a society with increasing prevalence of Alzheimer's disease and the associated dementia.

## Synthesis

Reviewing Editor: Laura Volpicelli-Daley, The University of Alabama at Birmingham Decisions are customarily a result of the Reviewing Editor and the peer reviewers coming together and discussing their recommendations until a consensus is reached. When revisions are invited, a fact-based synthesis statement explaining their decision and outlining what is needed to prepare a revision will be listed below. The following reviewer(s) agreed to reveal their identity: NONE.

I was unable to find reviewer's who were experts in Alzheimer's disease. Therefore, I reviewed the commentary myself although my area of expertise is alpha-synucleinopathies. Overall, I found the commentary well written. It summarized the current status of anti-amyloid beta immunotherapies. The tables were particularly helpful.

Just a few comments:

First, I am somewhat confused. A 30-55% slowing of cognitive decline seems good to me. I have colleagues who say the immunotherapies do not work. Would you be able to maybe provide a clearer statement about the extent of slowing of cognitive decline. For example, is there a slowing, but effect size of difference in MMSE scores are minimal? What specifically does the slowing of cognitive decline mean- slowed short term memory loss? Slowed spatial defects? Does the slowing mean that people can remain at their jobs longer or interact with grandchildren longer? As a scientist but also as someone with family history of Alzheiemr's disease, some more specific data with examples would be helpful and I assume the scientific and general community would find this helpful as well.

Could you mention a little more about biomarkers for preclinical/prodromal AD? Please be specific for amyloid beta and phospho-tau.

Could you be more specific about other treatments on the horizon? What small molecules are promising for AD? Please provide some more specific examples. What about anti-sense oligonucleotides?

Could you discuss the impact of the approval of subcutaneous injections for the economics of immunotherapies. Would this mean less time having to go to a hospital for infusions?
